# Pragmatic anesthetic approach for extracranial to intracranial bypass surgery in a patient with moyamoya disease and sickle cell disease: a case report

**DOI:** 10.1186/s13256-023-03857-6

**Published:** 2023-03-31

**Authors:** Shankar Lal, Vivienne Larney

**Affiliations:** Department of Anaesthesiology and Critical Care Medicine, Beaumont Hospital, Dublin, Republic of Ireland

**Keywords:** Moyamoya disease, Sickle cell disease, Extracranial to intracranial bypass surgery

## Abstract

**Background:**

Moyamoya disease is a chronic progressive cerebrovascular disorder. A proportion of 10–20% of patients with sickle cell disease have associated moyamoya disease and may require surgical revascularization as definitive treatment.

**Case presentation:**

A 22-year-old African lady with sickle cell disease and moyamoya disease, with extensive cerebral vasculopathy, was scheduled for elective extracranial to intracranial bypass surgery. She presented with right-sided weakness secondary to a hemorrhagic stroke of the left lentiform nucleus. She required a multidisciplinary team approach for preprocedural optimization. Her preoperative hemoglobin SS levels were reduced to less than 20%, with preoperative red blood cell transfusion to avoid sickling. We maintained normal physiology and optimal analgesia perioperatively. She was extubated after the successful surgical procedure and was transferred to Intensive care unit (ICU) for invasive monitoring, with subsequent discharge to the ward several days later.

**Conclusion:**

Optimal preprocedural optimization can decrease complications in patients with critically comprised cerebral circulation booked for extensive surgery such as ECIC bypass. We believe the presentation of anesthetic management of a patient with moyamoya disease and sickle cell disease may prove helpful.

## Background

Moyamoya disease (MMD) is a chronic progressive cerebrovascular disorder with obscure etiology. Unilateral or bilateral steno-occlusive changes occur, typically in the distal segment of the internal carotid arteries, with or without the association of the proximal segment of the anterior, middle and rarely the posterior cerebral arteries [1].

Medical treatment for MMD has failed to stop the progression of the disease. However, surgical revascularization effectively prevents ischemic attacks [2]. The presence of chronic medical conditions can worsen the prognosis of this serious condition. A proportion of 10–20% of patients with sickle cell disease (SCD) have associated MMD [3]. Patients with MMD associated with SCD present unique challenges to anesthesiologists. We wish to present a case of SCD, associated with MMD, for extracranial to intracranial (ECIC) bypass surgery and present the anesthetic management, which provided minimal disturbance to the critically compromised cerebral circulation during revascularization surgery.

## Case description

A 22-year-old African female was booked for extracranial to intracranial arterial bypass surgery due to extensive cerebral vasculopathy diagnosed at the age of 16 years. She had recently presented with a dense right-sided weakness due to a hemorrhagic stroke in the left lentiform nucleus. However, urgent neurosurgical intervention was deferred for several weeks due to her significant medical background, which warranted preprocedural preparation.

Her background was complicated with severe sickle cell disease associated with several vaso-occlusive crises and frequent blood exchange to keep the hemoglobin (Hb)SS < 20%, and to treat iron overload, extensive acute on chronic upper limb deep vein thrombosis (DVT), poor intravenous access, needle phobia, and severe depression. The agreed perioperative plan was followed by the multidisciplinary team comprising anesthesia, hematology, and neurosurgery. She received a preoperative red blood cell transfusion in the referring hospital 4 days before the procedure. On admission to our neurosurgical center, her baseline Hb was 11.9 g/dl, her HbSS fraction was 10%, and her baseline neurological examination revealed a Glasgow Coma Scale (GCS) of 15/15, with a mild right hemiparesis. All her other baseline investigations, including electrocardiogram (ECG), echocardiogram (ECHO), and chest X-ray, were normal. She received intravenous fluids preoperatively to maintain adequate hydration, along with routine medications until the procedure. Her routine medications included deferasirox, folic acid, pregabalin, and vitamin D3. Her aspirin had been on hold since her hemorrhagic stroke.

We ensured the availability of antibody-free packed red blood cells (PRBCs) before induction of anesthesia. After applying the Association of Anaesthetists of Great Britain and Ireland (AAGBI) standard monitoring, an arterial line was inserted awake, following which anesthesia was induced with propofol, fentanyl, and rocuronium after preoxygenation. The trachea was intubated, and peripheral large-bore intravenous access was obtained. She had a peripherally inserted central catheter (PICC) line *in situ*. We avoided central venous cannulation due to her history and increased risk of deep venous thrombosis. The patient underwent a left ECIC bypass from the superficial temporal artery to the middle cerebral artery, and anesthesia was maintained with sevoflurane in oxygen and air. She received a target-controlled infusion of remifentanil. A phenylephrine infusion was used, targeting a mean arterial pressure (MAP) of 80 mmHg (10–20% above her baseline) during the procedure. Her ventilation, oxygenation, temperature, and blood pressure were maintained within normal limits throughout the procedure. She received heparin during the surgery, which did not require reversal with protamine at the end of the procedure. The procedure was performed successfully with minimal blood loss, and she did not require a transfusion. She was successfully extubated and transferred to the postanesthesia care unit. Her postoperative neurological examination was similar to her preoperative baseline. She was transferred to the intensive care unit for invasive blood pressure monitoring postoperatively and was discharged to the ward several days later. She received her routine medications soon after the procedure, and we ensured adequate postoperative analgesia and hydration.

## Discussion

MMD is a rare progressive cerebrovascular disease of unknown origin. It affects the large intracranial arteries with secondary development of collaterals, which present as a “puff of smoke” on CT angiogram—this is where its name, moyamoya, comes from (a “puff of smoke” in Japanese) [4]. MMD can occur in children and adults. The common presentations are headaches, transient ischemic attacks (TIAs), ischemic or hemorrhagic strokes, and seizures [5]. However, intracranial hemorrhage is seven times more likely to occur in adults. Our patient presented with a hemorrhagic stroke in the left lentiform nucleus. MMD is associated with various other diseases: Down’s syndrome, cranial therapeutic irradiation, neurofibromatosis type 1, and sickle cell disease, with a prevalence of 10–20%. In addition, renal artery stenosis, giant cervicofacial hemangiomas, hyperthyroidism, and congenital cardiac anomaly are rarely associated with MMD. Our patient had sickle cell disease [5].

Pharmacological treatment with oral antiplatelets, calcium channel blockers, steroids, and anticonvulsants has failed to stop the progression of moyamoya disease effectively [6]. However, it can be considered in patients with cerebral ischemic disease where surgical treatment is deemed too high risk.

Surgical revascularization successfully augments cerebral flow and reduces the risk of ischemic stroke [7]. Our patient required direct revascularization between the left superficial temporal artery and the middle cerebral artery. Indirect revascularization procedures, such as encephalodurarteriosynangiosis, encephaloduroarteriomyosynangiosis, and pial synangiosis, have been performed with various outcomes [8].

The perioperative management of patients with moyamoya disease associated with sickle cell disease presents several unique challenges. The common ones are the high risk of vaso-occlusive crisis, cerebral ischemic events, and the existence of chronic pain, along with alloimmunization due to frequent blood transfusions [9]. Intraoperatively, maintenance of cerebral perfusion is dependent on stable hemodynamics and the maintenance of blood pressure within 10–20% of the patient’s baseline. Additionally, avoidance of hypercarbia, hypocarbia, hypoxemia, acidosis, and dehydration, which predispose the patient to sickling, are critical steps during the procedure. Multimodal analgesia should be considered for managing postoperative pain, with a low threshold for opioid administration due to greater tolerance.

## Conclusion

A pragmatic multidisciplinary perioperative approach can decrease the risk of cerebral ischemia and sickling in patients with sickle cell disease associated with MMD during and following revascularization surgery. Preoperatively, red blood cell transfusion may be required, targeting a HbSS fraction of less than 20–30%. Intravenous fluids should be administered preoperatively to avoid dehydration, which predisposes the patient to sickling. Intraoperatively, the maintenance of normal physiological parameters, adequate hydration, adequate analgesia, and vigilant monitoring results in good outcomes.

## Data Availability

Not applicable.

## Abbreviations

MMD: Moyamoya disease
SCD: Sickle cell disease
ECIC: Extracranial to intracranial
TIA: Transient ischemic attack
